# Beyond pain: a study on the variance of pain thresholds within BDSM interactions in dominants and submissives

**DOI:** 10.1192/j.eurpsy.2022.718

**Published:** 2022-09-01

**Authors:** E. Wuyts, N. De Neef, V. Coppens, A. Schuerewegen, I. De Zeeuw-Jans, M. Van Der Pol, M. Morrens

**Affiliations:** 1 Collaborative Antwerp Psychiatric Research Institute (CAPRI), Faculty Of Medicine And Health Sciences, Campus Drie Eiken, University Of Antwerp, Edegem, Belgium; 2 Europe Hospitals, Campus St Michel, Brussels, Belgium; 3 University Hospital Antwerp, University Forensic Center, Edegem, Belgium; 4 VZW Club 78, Fetish Club 78, Ham, Belgium

**Keywords:** BDSM, pain thresholds, sexuality, pain cognitions

## Abstract

**Introduction:**

BDSM is an acronym describing bondage & discipline, dominance & submission and sadism & masochism. Afflicting or receiving pain is usually an important part of the BDSM interaction.

**Objectives:**

This research focuses on better understanding the aspect of pain within a BDSM interaction by investigating the differences in 1) baseline pain thresholds, 2) the impact of a BDSM interaction on those thresholds and 3) threshold moderating factors like pain cognition between submissive and dominant BDSM participants and control individuals.

**Methods:**

Submissive and dominant counterparts of 35 couples were recruited to participate in a BDSM interaction, of which 34 dominants and 33 submissives were included in analyses. A non-BDSM interested control group (n=27) was included to control for social interaction, of which 24 were included in analyses. Pain threshold measurements were measured at three points in time and pain cognitions scales were taken.

**Results:**

BDSM practitioners have a higher pain threshold overall and a BSDM interaction will result in a temporary elevation of pain thresholds for submissives. Additionally, pain thresholds in dominants will be dependent upon their fear of pain and tendency to catastrophize pain and submissives will experience less fear of pain than the control group.

**Conclusions:**

This study helps shed further light on the biological processes behind a BDSM interaction through pain threshold measurements. By enhancing our understanding of the mechanisms behind a BDSM interaction in this way, we aspire to relieve the stigma these practitioners still endure.

**Disclosure:**

No significant relationships.

